# Supplementary material for: IL-33 priming and antigenic stimulation synergistically promote the transcription of proinflammatory cytokine and chemokine genes in human skin mast cells
Source: BMC Genomics. 2023 Oct 6;24:592. doi: 10.1186/s12864-023-09702-w (PMC10557204; doi:10.1186/s12864-023-09702-w)
Supplement: Supplementary file 7 — Additional file 7: Figure S1. K-means clustering elbow plots. Figure S2. Differential mRNA expression analysis of RNA-seq prepared from HSMCs received various combined treatments. [file 12864_2023_9702_MOESM7_ESM.pdf]

Figure S1

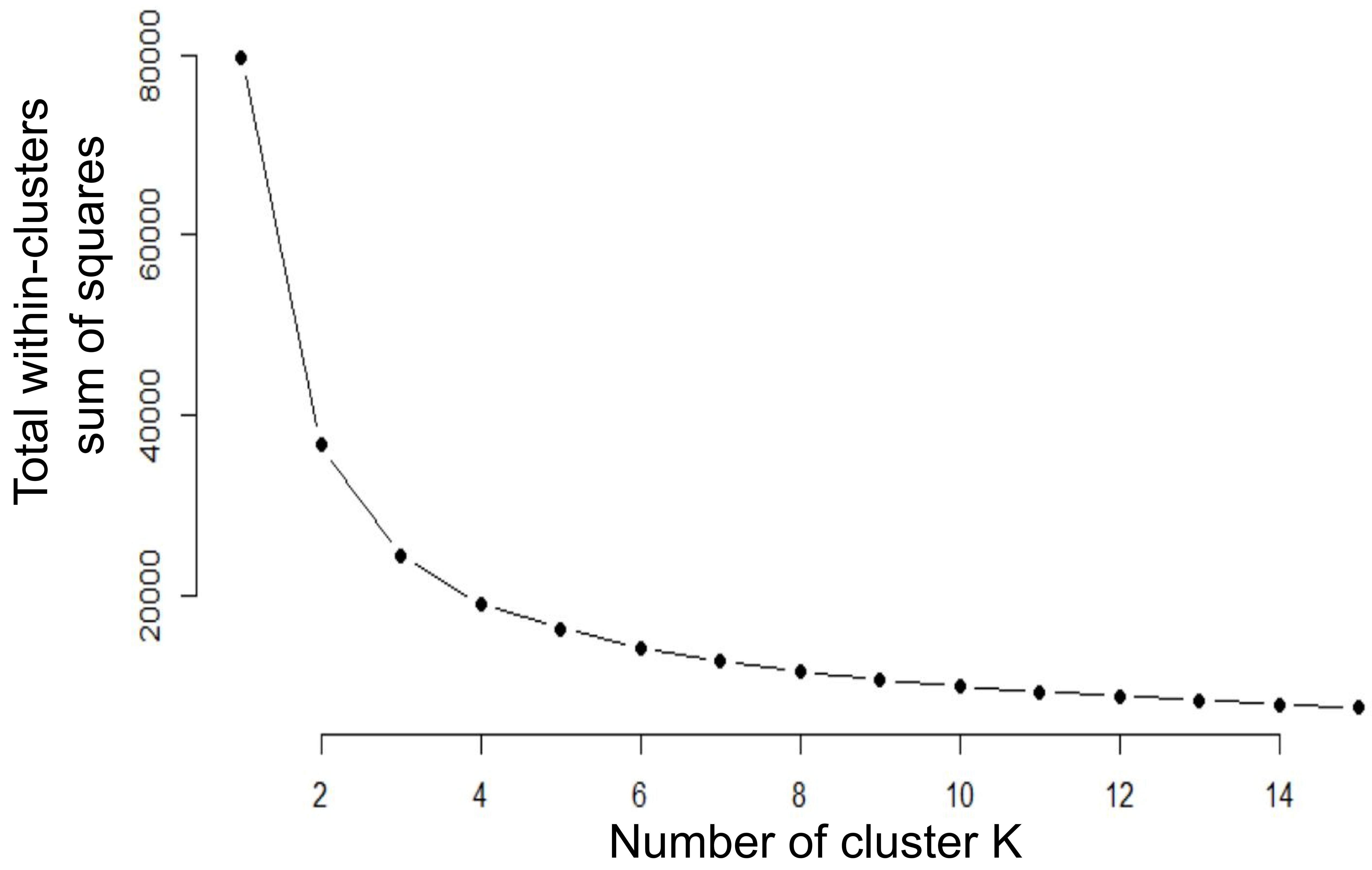

Figure S1. K-means clustering elbow plots. The optimal numbers of K in the RNA-seq datasets (two biological samples) were determined using R packages Tidyverse (version 1.3.1). The within cluster sum of squares decrease with the increment of clusters number, and the optimal number of clustering was selected at the last one significantly reduced the within cluster sum of squares (at the inflection point of the curve). The optimal clustering was at number of six for the RNA-seq datasets.

Figure S2

A

IgECL vs IgECL + IL-25

Gene	E	E + 25	LogFC(E+25/E)
PDIA3P2	25.8	74.2	1.5
FSTL1	10.1	24.3	1.3
TFP12	16.9	37.8	1.2
MMP3	13.3	28.7	1.1
RARRES2	11.5	24.8	1.1
CEMIP	15.9	34.1	1.1
IGFBP3	11.2	23.6	1.1
ADAMTS1	17.8	37.4	1.1
RCN3	12.1	25.3	1.1
NT5E	11.8	24.3	1.0
CDH11	12.8	26.2	1.0
PPIAP11	10.5	21.6	1.0
IL6	54.6	111.4	1.0
NNMT	40.5	82.0	1.0
MMP1	95.0	192.3	1.0
ITM2C	15.6	31.4	1.0
MEDAG	19.8	38.7	1.0
TMEM176B	13.7	26.4	1.0
COL6A1	58.1	111.7	0.9
SPARC	39.6	76.1	0.9

B

IgECL vs IgECL + TSLP

Gene	E	E + T	LogFC(E+T/E)
COX7CP1	13.4	31.1	1.2
RMRP	29.3	58.8	1.0
PLXNA3	11.4	22.1	1.0
RPS28P7	32.7	61.6	0.9
SELENOKP1	12.9	23.3	0.9
BORCS8	11.0	18.0	0.7
MAPK8	13.2	20.8	0.7
PDIA3P2	41.8	65.5	0.6
SNHG19	14.9	23.3	0.6
NUFIP2	13.9	21.4	0.6
SNHG9	15.9	23.6	0.6
SMIM14	10.3	15.1	0.6
RPL9P32	22.5	33.0	0.5
PFN1P1	35.6	52.0	0.5
PTMAP4	17.2	25.0	0.5
ELOVL6	35.7	51.2	0.5
UBES2	41.4	59.0	0.5
MAP4K3	15.9	22.6	0.5
ECHDC1	12.4	17.7	0.5
SERGEF	12.7	18.1	0.5

C

IgECL+IL-33 vs IgECL+IL-25+IL-33+TSLP

Gene	E	E + 33	E+25+33+T	LogFC(E+25+33+T/E+33)
EED	9.0	10.2	19.8	1.0
RF02196	61.8	25.8	49.8	0.9
RHOH	47.1	20.4	38.9	0.9
OSM	837.8	240.2	435.7	0.9
SOCS2	209.4	60.1	106.5	0.8
MIIP	27.5	34.7	58.8	0.8
HBEGF	15.7	10.4	17.6	0.7
LYPLA1	51.2	34.1	56.8	0.7
IL1RN	128.5	152.3	251.9	0.7
EMP1	303.0	184.1	299.6	0.7
ETS2	48.1	28.8	46.7	0.6
ERI1	35.3	16.1	25.4	0.6
IL2RA	7.9	20.3	31.1	0.6
IVNS1ABP	45.2	113.0	170.6	0.6
EFCAB13	26.4	34.4	51.7	0.6
RBM17	64.7	85.2	126.4	0.6
MYRIP	14.9	10.7	15.8	0.6
TMEM173	48.6	39.8	58.8	0.6
NT5DC2	4.2	12.0	17.5	0.5
MRPL48	14.0	10.9	15.9	0.5

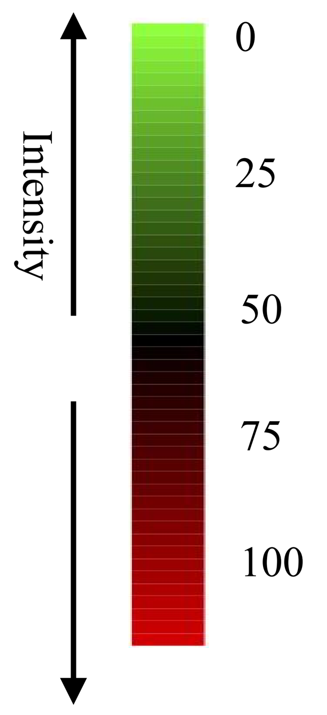

Figure S2. Differential mRNA expression analysis of RNA-seq prepared from HSMCs received various combined treatments. Heatmap representations of top ranked genes after the combined IgECL+IL-25 treatment (A), IgECL+TSLP treatment (B) or IgECL+IL-33+IL-25+TSLP treatment (C). Legends: E, IgECL; E+25, IgECL+IL-25 treatment; E+T, IgECL+TSLP treatment; E+33, IgECL+IL-33 treatment; E+25+33+T, IgECL+IL-25+IL-33+TSLP treatment; LogFC, log2 fold change. The numbers indicate RNA reads (RPKM). Data A-C represent two biological samples.
